# Supplementary material for: Individual participant data network meta-analysis of psychosocial interventions for survivors of intimate partner violence: Study protocol
Source: PLoS One. 2025 Mar 18;20(3):e0306669. doi: 10.1371/journal.pone.0306669 (PMC11957554; doi:10.1371/journal.pone.0306669)
Supplement: Attachment — Submitted filename: Response to Reviewers.docx [file pone.0306669.s002.docx]

Dear Dr Leight and esteemed peer-reviewers,

Firstly, we would like to thank you for the thorough review and insightful comments, we are positive that addressing them will improve the manuscript and our proposed study. In the following, we address the comments one by one:

#Reviewer 1

***ABSTRACT*** *This is clear but there are some grammatical errors and missing words. The word "possibly" is rather casual for a protocol and I suggest setting out in a formal manner what the authors propose to do and what caveats may prevent their plans' realisation.”*

We thank the reviewers for this sharp observation. The word “possibly” has been removed, and the planned actions are set out more clearly: “We anticipate that not all study authors will provide access to IPD, which is a limitation of IPDNMA. We aim to address this by combining studies with aggregate data and studies with IPD using Multi-Level Network Meta-Regression (ML-NMR) implemented in multinma. “ We have gone through the manuscript carefully and corrected grammatical errors.

***Introduction***

*“There are some vague expressions: "involved in" IPV, "intertwined with the course of mental distress", "do not use optimal design", "all necessary information", "blurs the evidence", "have effects up to a certain extent". Could the authors be more specific? Intertwined how? Which designs and information are lacking? In what way is the evidence "blurred"? What effects? What extent?”*

Thanks for picking this up. We have checked the manuscript carefully and clarified these expressions to be more specific, and have elaborated on all the aforementioned points: “both men and women victims/survivors and perpetrators of IPV”, “The course of IPV affects the course of mental distress and vice versa”, “the most methodologically robust design of a randomised and controlled trial”, “the descriptive statistics that are necessary for a meta-analysis for all the outcomes”, “heterogeneity is an important limitation for these outcomes too”, “. they reduce physical and psychological IPV, but do not reduce sexual IPV and overall IPV”.

*“What is meant by ecological bias here?”*

We thank the reviewer for giving us the opportunity to highlight a strength of this study. An explanation was added: “Meta-analyses of aggregate data are limited to the outcomes reported in publications and can only explore study-level moderators which often lack statistical power and are vulnerable to ecological bias, where covariate effects at the study-level do not accord with those seen at the individual level”.

*“P4 line 91 "To provide reliable and robust estimates of interventions" - this is a bit vague. Estimates of intervention what - efficacy? In what way will the results be reliable and robust? Perhaps more specific language related to the methods would be more appropriate e.g. more precise? More specific?”*

Thank you for pointing this out. We have re-worded the statement of the aims to be clearer: ”This study aims to provide more valid and detailed estimates of the clinically relevant benefits and harms than the existing pairwise meta-analyses, as well as moderators to that, of each type of psychosocial intervention comparatively by obtaining the IPD of the studies to enable exploration and adjustment of effect modifiers. We will fulfil this aim by conducting an IPDNMA, to estimate the relative effectiveness of multiple psychosocial interventions for mental health, psychosocial well-being, IPV, safety behaviours, intervention dropout and harms (i.e. adverse effects of the interventions. We also aim to estimate study-level and individual-level moderator effects to identify which psychosocial intervention suits individual IPV survivors best.”

*“Acceptability is grouped together with efficacy but its measurement is likely to be much more heterogeneous than for efficacy; the authors have not commented on this or said what they mean by the term when used quantitatively. Have previous studies been able to apply NMA to an outcome like acceptability?
Likewise, how will safety be conceptualised quantitatively?”*

We thank the reviewer for pointing this out. Yes, previous NMAs of psychotherapy have had acceptability as an outcome, but they operationalised it as all-cause dropout, due to heterogeneity in reporting ([Cuijpers et al, 2019](https://jamanetwork.com/journals/jamapsychiatry/fullarticle/2730724); [Cuijpers et al, 2021](https://onlinelibrary.wiley.com/doi/full/10.1002/wps.20860)). We hope that with the IPD we will be able to overcome that for at least some studies. We will use intervention dropout as the closest available quantitative indication of acceptability, as opposed to study dropout, because we believe that dropping out from research assessments is different from skipping intervention sessions, and the former has hardly, if any, clinical relevance. Given that intervention dropout is merely an indication of acceptability and it might not accurately reflect it, we now refer to intervention dropout as our study outcome instead of intervention acceptability.

We now refer to reduction in IPV and increase in safety behaviours as our study outcomes, instead of “safety” only, to avoid confusion:

“Which victim/survivor and intervention characteristics moderate the comparative effectiveness of each set of intervention components that have been tested together in reducing IPV and improving safety behaviours of IPV victims/survivors?”

“Which victim/survivor and intervention characteristics moderate the dropout from each intervention?

*“P4 RQ2 - while I assume specific mental health conditions will be named in the methods it would help to be more specific about the mental health outcomes of interest. Common mental disorders? Severe mental illness? Any psychiatric diagnosis?”*

We thank the reviewer for this sharp observation. We are interested in all mental health-related outcomes, but most often this is symptoms of depression and/or anxiety, post-traumatic stress, and substance use, therefore we set these as our primary outcomes. We anticipate that there will be too scarce evidence available for other outcomes to support any analysis. We now specify that from the research question already: “Which victim/survivor and intervention characteristics moderate the comparative effectiveness of each set of intervention components that have been tested together in improving commonly tested mental health and psychosocial well-being outcomes of IPV victims/survivors?”

*“The RQs switch between "efficacy" and "effectiveness"; I suggest consistency throughout.”*

We thank the reviewer for this comment. We have now consistently used “effectiveness” throughout the manuscript. We will include all RCTs comparing psychosocial interventions with a standard care control condition or another psychosocial intervention, and the majority of included studies will be effectiveness studies, so this is the most appropriate term.

*“Given the quantitative methods planned, it seems appropriate to present hypotheses, either in addition or instead of RQs.”*

We understand this comment and we thank the reviewer, however meta-analyses are retrospective explorative investigations. Hypothesis are more common in prospective experimental designs. We have not made any changes in the text based on this comment.

***METHODS***

*“Line 110 - harms have not previously been mentioned unless this is in reference to "safety". Perhaps the authors can define what they mean by both. Without clarity I would assume that safety refers to whether women report fewer IPV incidents following intervention whereas harms would be adverse effects of the intervention.”*

We thank the reviewer for giving us the opportunity to make this important distinction. The harms are not related to safety, they are adverse intervention effects. They are now mentioned and referred to as “adverse effects if the interventions”.

*“Line 115 it would be better to mention the relationship between this and the Micklitz study in the Introduction and explicitly state what that study found and what gaps this study will fill.”*

We thank the reviewer for this suggestion, we have now added a paragraph in the introduction, right before the aims: “IPDNMA has the potential to address many of the limitations and issues raised by one of the most recent meta-analyses with a very close scope to this one (16). Micklitz and colleagues found that psychosocial interventions, and especially interventions combining advocacy with psychotherapy, i.e. integrative interventions, had a small yet statistically significant effect in reducing depression and post-traumatic stress symptoms, compared to waiting list or inactive control conditions. However, there was considerable heterogeneity even in the subgroup analyses, and half the studies could not be included in the quantitative meta-analysis because the necessary statistics were not reported at the publications of the primary studies (16). In addition, the authors highlighted the need to test participant-level moderators of effect (16). We aim to build on the comprehensive scope and rigour of the study by Micklitz and colleagues by updating and adapting their searches, and seeking IPD from the included studies. Given that most RCTs have used Treatment As Usual (TAU) control conditions, or participants were most commonly allowed to seek other help, and also that we are going to compare interventions, we are going to estimate the effectiveness of the interventions, i.e. compared to a real-world standard, rather than efficacy, i.e. that the interventions have some effect compared to nothing, which is usually a sterilised control condition.”

*“Table 1 - it is not clear why psychological and physical risk factors are mentioned in the description of eligible interventions.
The Outcomes box introduces well-being and quality of life outcomes but these constructs are not discussed in the Introduction. Safety and acceptability outcomes are not mentioned in this box but feature in the RQs.”*

We thank the reviewer for this suggestion. The text on psychological and physical risk factors has been removed to avoid confusion, and explanations have been added to the “Participants” and “Outcomes” sections of the table:

**Table 1. Eligibility criteria by PICO**

| **Participants** | Individuals or couples reporting either past or ongoing IPV at randomisation, but studies solely on those involved in perpetration are excluded. Studies are deemed partially eligible if they did not recruit participants based on IPV status, but they measured IPV at baseline, and a proportion of the participants had been victimised at baseline. The IPD only of the victimised participants will be requested. If it is not possible to obtain the IPD of a partially eligible study, and outcome measures separately for victimised participants are not reported in the publication, the study will not be included in the analysis at all. |
| --- | --- |
| **Interventions** | Psychosocial interventions aimed at IPV survivors using any mode of delivery at any setting. Both psychotherapeutic and advocacy interventions are deemed as psychosocial. Interventions aimed solely at perpetrators, primary prevention to individuals who have not experienced IPV at baseline, and physical safety (such as being in a refuge/shelter and police interventions) are not included. |
| **Comparators** | All comparators are eligible, including no intervention, waiting list, Treatment as Usual (TAU), another active intervention, placebo or sham version of the intervention. The interventions will be categorised and classified into nodes of the network based on their description regardless of whether they were tested as control or active condition in the original studies. |
| **Outcomes** | Eligible outcomes are all outcomes related to mental health (e.g. depression) as well as psychosocial outcomes (e.g. empowerment and social support) . Studies measuring only IPV are not eligible, but if the included studies have measured IPV, it will be analysed. Likewise, studies are not included based on whether they measured intervention dropout, and health service use, but these outcomes will be analysed. |

*“Line 128 - perhaps it would be clearer to say that "their data will be analysed together with" the other studies rather than "they will be merged with"?”*

We thank the reviewer for this suggestion. The text was rephrased accordingly: “Only randomised controlled trials (RCTs) are eligible for inclusion, including pilot RCTs and studies with few participants, as in most cases they will be analysed together with larger studies testing a similar intervention.”

*“Line 133 - given that the purpose of this article is to publish the study protocol I do not think it is enough to refer readers to the Appendix and a different paper for the search strategy. At a minimum I would expect all the search terms to be named.”*

We thank the reviewer for this important addition. The full search string was added to the manuscript as a table.

intimate partner violence OR spouse abuse OR domestic violence OR gender-based violence OR rape OR battered women OR "intimate partner violence" OR "partner violence" OR "interpersonal violence" OR "dating violence" OR "domestic violence" OR "family violence" OR "gender-based violence" OR "gender based violence" OR "violence in close relationships" OR "intimate partner abuse" OR spous* abuse OR "abusive relationship" OR "marital abuse" OR "marital rape" OR "dating abuse" OR "intimate partner aggression" OR "battered women" OR "battered wives" AND Mental health services OR Psychotherapy OR Couples Therapy OR Counselling OR Psychosocial intervention OR Social Work, Psychiatric OR Psychiatric Rehabilitation OR Therapy, computer-assisted OR Telemedicine OR Internet-Based Intervention OR Distance counselling OR "intervention" OR "treatment" OR "program" OR "therap*" OR "psychotherap*" OR "counsel*" OR "support service*" OR "advocacy" OR "secondary prevention" OR "tertiary prevention" OR "rehabilitation" AND randomized controlled trials as topic OR clinical trials as topic OR "randomized controlled trial" OR "clinical trial" OR "controlled clinical trial" OR "clinical trial" OR "clinical trial protocol" OR "clinical study" OR RCT OR random* OR trial

*“Line 137 - forward and backward citation tracking?”*

We thank the reviewer for this opportunity to clarify. An explanation was added to the manuscript: “the reference lists of the included studies will be checked for eligible papers, as well as which studies cite the eligible studies”.

*“Line 174 - again, it is not enough to refer readers back to the Micklitz paper. At this point the reader is interested to know which data will be extracted or requested. This is especially important for the RQs on efficacy, effectiveness, acceptability, safety and harms. I note the later section on Outcomes; perhaps these can be combined?”*

We thank the reviewer for this comment. The data that are extracted for this study are listed in the paragraph “Data extraction of study characteristics and aggregate data: “The study characteristics to be extracted in a spreadsheet are: author, publication year, citation, country, recruitment setting, trial setting, number of trial arms, number of follow-ups, timing of follow-ups, timing of post-treatment, primary and secondary outcomes, type of randomisation, unit of allocation, method of randomisation, sample size in total and in each arm, treatment and study dropout, intention-to-treat or per-protocol analysis, age, gender, current frequency and/or severity of IPV, past frequency and/or severity of IPV, specific target population, eligibility criteria, description of intervention, theoretical orientation, delivery mode, type of therapists/facilitators, number and duration of sessions, frequency of sessions, description of control, whether adverse events and adherence to the intervention were reported, conclusions, the means and standard deviations of all relevant outcomes at all timepoints, or which statistics were reported instead of mean and standard deviation.”

We do not refer to the Micklitz paper to report which data are extracted, we are simply explaining that we will make use of the data Micklitz and colleagues have already extracted for the studies they included, instead of extracting them again. The text had been rephrased to clarify: “The characteristics and aggregate data that have already been extracted by Micklitz et al (16) for the studies they included in their meta-analysis will be used in our analysis.”

We believe we cannot combine this section with the “Outcomes” sections, because we think we should report on all the study characteristics extracted, and not just the outcomes. Data extraction is an integral part of every systematic review, so we think it merits being an independent section.

*“Line 197 - it is not clear why not reporting one characteristic might intrinsically jeopardise the quality of an included study.”*

We thank the reviewer for this insightful observation. Indeed, not reporting on some characteristics does not intrinsically does not compromise the quality of the trial, but it does make it more difficult to adequately assess the quality of the trial. The text has been slightly rephrased from “quality assessment” to “ assessment of certainty in the evidence”, also because this refers to the GRADE assessment, and GRADE assesses certainty in the evidence.

*“Line 215 - "in a sort of "within-study synthesis"" is rather casual phrasing for a protocol. Can the authors describe precisely what they propose to do?”*

We thank the reviewer for this comment. The sentence has been rephrased and a reference has been added: “If a study uses more than one scale for the same outcome, these scales will be combined in a “within-study-synthesis”, by using meta-analytical methods (39).”

*“Given that the authors speak about "psychosocial" interventions in the Introduction I wonder whether this term should be defined there, perhaps making reference to the Micklitz taxonomy there instead of late in the Methods.”*

We thank the reviewer for this observation. We now define the different types of psychological interventions in the introduction too: “Micklitz and colleagues categorised the tested psychosocial interventions into three main categories: psychological, advocacy-oriented and integrated (a combination of the two) (16). “

*“Line 253 does mindfulness not come under third wave therapies?”*

1. We thank the reviewer for noticing this inconsistency. Mindfulness is now mentioned only under third wave therapies. “Third wave therapies: psychological interventions with more targeted cognitive approaches, such as mindfulness

Systemic interventions: family or couple based psychological interventions

Other psychological interventions: other psychological approaches tested by a small number of studies, such as interpersonal, humanistic or meditation based”

*“Line 269 "which is not ideal" - this is vague. Can the authors specify the problems with heterogeneous outcome measures?”*

We thank the reviewer for this suggestion. We have rephrased and specified why heterogeneity in outcomes is problematic: “heterogeneity in the estimation of effectiveness of interventions, and thus limits certainty, hampers interpreting the estimates accurately and establishing clinical relevance”

*“The outcomes section demonstrates that the focus is on common mental disorders, only. I suggest clarifying this throughout the manuscript and justifying at some point why other disorders are not included.”*

We thank the reviewer for giving us the opportunity to clarify. The reason why we do not mention symptoms of more severe disorders is because they have rarely ever been measured by the RCTs, so there is not enough evidence to conduct quantitative synthesis. Other mental health related outcomes, such as self-efficacy, are mentioned in the manuscript as secondary outcomes, because they are less commonly measured, as reported in the meta-analysis by Micklitz and colleagues, but there will be enough studies to allow for evidence synthesis. This clarification has been added to the manuscript: “in our study, the primary outcomes are the most commonly measured mental health outcomes, as the results of the Micklitz et al meta-analysis showed that there is not sufficient evidence on other outcomes to allow for evidence synthesis.”

*“Line 277 - Certain outcomes may be less well known eg space for action. If this is a construct with a validated scale, perhaps the authors can indicate the most widely used in brackets?”*

We thank the reviewer for this observation. We removed this outcome, because indeed it has not been defined this way by any RCT.

*“Given the range of outcomes to be collected I suggest that the RQs be rewritten to reflect the different sub-groups of outcomes.”*

We thank the reviewer for this remark, the research questions now include all sets of outcomes and they are organised by outcome:

“1.Which victim/survivor and intervention characteristics moderate the comparative effectiveness of each set of intervention components that have been tested together in improving commonly tested mental health and psychosocial well-being outcomes of IPV victims/survivors?

2. Which victim/survivor and intervention characteristics moderate the comparative effectiveness of each set of intervention components that have been tested together in reducing IPV and improving safety behaviours of IPV victims/survivors?
3. Which victim/survivor and intervention characteristics moderate the comparative effectiveness of each set of intervention components that have been tested together in improving service use of IPV victims/survivors?
4. Which victim/survivor and intervention characteristics moderate the dropout from each intervention?”

*“Line 306 - "as demonstrated by (42)" is not enough. Please state what will be done.”*

We thank the reviewer for pointing this out. The comment no longer applies, because we decided against using this method. We now state what exactly will be done: “Given that numerous potential moderators are likely to be available, we will explore if it is possible to reduce the number of covariates by testing which are highly correlated, and running collinearity diagnostics”

*“Line 309 - likewise, "as described in (43)”*

We thank the reviewer for pointing this out. We now give a summary of the method presented at the paper of Papadimitropoulou et al: “If the regression coefficients and descriptive statistics for background characteristics are available in the publications of studies that do not provide IPD, we will create pseudo-IPD by simulating from a model using the reported baseline summaries and regression coefficients for these effect modifiers”.

*“Line 316 - "whether... are effect modifiers or prognostic factors": can the authors clarify whether prognostic factors are identified differently to effect modifiers I.e. how this is determined statistically?”*

We thank the reviewer for giving us the opportunity to clarify. We have clarified that we will use the results from the IPDMA where covariates*treatment interaction effects will be inspected to assess whether a covariate is an effect modifier or a purely prognostic factor. “We will assess whether survivor and setting characteristics (such as being separated or still living together with their partner, and clinical or community settings) are effect modifiers or prognostic factors, based on results of the IPDMA (i.e. whether there are interactions between intervention and characteristics)”.

*“Line 322 - again, please elaborate on the Methods rather than referencing study (44).”*

We thank the reviewer for giving us the opportunity to clarify. We have now added the method used: “ML-NMR is a method that can combine studies with IPD and studies with aggregate data summaries by defining a participant-level regression. Studies with IPD directly inform the individual-level model, whereas studies with aggregate data studies inform an integral over the model based on the covariate distribution of each study”

*“Line 323 - this is the first time acceptability is defined as the dropout rate. I suggest this needs to be stated in the Introduction to help readers to understand the RQs.”*

We thank the reviewer once again, as previously mentioned we now use the term “intervention dropout” instead of acceptability throughout the manuscript.

*“Line 335 - "will be compared" - how? Statistically? In some other way?
I am not a statistics expert but am interested whether corrections are needed for multiple comparisons, based on the number of analyses planned. These may be built into the NMA programme but it would help readers (few of whom will be experts) to be as clear as possible about how the analyses work.”*

We thank the reviewer for giving us the opportunity to clarify. We have deleted this sentence, as the focus of the work is not on comparing different models, but on estimating the effects of multiple interventions and moderator effects. Whilst we are focusing on estimation rather than on hypothesis testing, the reviewer raises an important point that this means that we are interpreting multiple effect estimates. Caution is therefore needed in the interpretation as with so many estimates to interpret some apparent effects may be “chance-findings”. We have added a sentence to note that we will be appropriately conservative in interpreting the results: “Given the sheer number of intervention and moderator effects that we will estimate, we acknowledge that the probability of chance findings increases. Therefore the results will be interpreted appropriately conservatively.”

***DISCUSSION***

*“Line 341 - I am not sure the planned collaboration with authors needs to be repeated in the Discussion. It would be more helpful to link the planned work to gaps in the literature.”*

We thank the reviewer for this insightful remark. We still believe the collaboration is a distinctive key element of this study, so we would prefer to keep it in the discussion, even though we have reduced the amount of text devoted to it, and we have added more on the gaps this work is going to fill: “We intend to fulfil this aim by establishing a collaboration with the authors of the RCTs. This work will disentangle a considerable part of the heterogeneity in the field of psychosocial interventions for IPV victims/survivors. More specifically, the moderating effects of key participant characteristics will be investigated, which thus enhances the evidence on whether intervention effectiveness differs for survivors with different and perhaps intersecting identities. Moreover, we will examine more thoroughly which components of the interventions precisely are of particular importance.”

*“Line 349 - GRADE is presented here as quantifying certainty but in the Methods as determining quality. I suggest consistency of terms throughout.”*

We thank the reviewer for pointing this out, we have now used “certainty in the evidence” throughout the manuscript.

*“Line 353 - "some nuancing might be missed" - this is vague. Can the authors specify the potential problems of combining different measures?”*

We thank the reviewer for this opportunity to clarify. We have now specified in the text that if a measure that is rarely used captures a certain aspect of the concept measured, and this is not captured by the more commonly used measures, this aspect will be missed: “In addition, it may not be possible to harmonise outcomes across studies that have measured them in different ways, which means that some facets of the concepts included only in measures rarely used might be missed. We plan to address as much as possible by sensitivity analyses.”

*“Line 356 - my understanding of the Methods was that the Micklitz search would be repeated and not expanded. Can this be clarified and if expanded, please describe how under Methods.”*

We thank the reviewer for pointing this out. We now clearly explain both in the methods and discussion that we expand the search by including searching the Web of Science database: “This search strategy was used by Micklitz and colleagues, so an update of their search will be performed in the databases they searched (PsycINFO, Medline, Embase, and Cochrane Central), and additionally on Web of Science.”

*“Line 359 - "the field of IPV is highly heterogeneous" - what does this mean? Existing research uses diverse methods and measures? Or IPV affects diverse pateints? Please clarify.”*

We thank the reviewer for this opportunity to clarify. We now clarify that we consider the field of IPV heterogeneous for both these reasons: “The field of IPV is highly heterogeneous, both because IPV affects diverse individuals and because the existing research has tested various interventions and outcomes with different measures, so matching individuals and contexts with interventions is crucial.”

**#Reviewer 2**

*“l 24 explain this sentence more,as an oversimplification”*

We thank the reviewer for this remark. The sentence has now been rephrased to: “The course of IPV affects the course of mental distress and vice versa”.

*“L28 instead of the word current put a date identifier so future readers do not have to check the publication date to understand the context”*

We thank the reviewer for this insightful observation. We now state that the cost of living crisis since early 2022: “Therefore, it is likely to continue rising during the cost of living crisis starting in early 2022”

*“Lines 38-43 need expansion - it's not just heterogenous for example but failure to analyse subpopulations, and also for example in some groups the follow up periods are particularly important. The current statements are so generalised as to be unhelpful and almost minority-blind.”*

We thank the reviewer for pointing this out. We have now specified in the text the different groups, with emphasis on minorities: “This is particularly problematic, because the survivors are indeed quite diverse, and the experiences of minoritised groups that face discrimination are different (19), and come from different contexts (e.g. staying with the perpetrator, staying in a shelter, co-parenting with the perpetrator) indicating different needs from psychosocial support”.

*“L 55 instead of 'was not examined quantitatively' point out that the theoretical approach used - eg realist review - was not appropriate for quantitative evaluation - as it currently reads as a criticism of the previous works rather than a critique”*

We thank the reviewer for pointing this out. We have now rephrased to acknowledge that lack of quantitative analysis was not appropriate for this study: “However, since this study was a realist review, it was not appropriate to examine heterogeneity quantitatively”.

*“L82 should be expanded on in the limitations in the discussion as also the issue of not getting data - emphasising the considerable efforts being made to do so - yes the authors do include this risk but they need to consider the implications more as this is a major issue with this method. This would also give them the chance to emphasise their good overall design to mitigate and compare.”*

We thank the reviewer for this observation. We can never be sure that we have captured and adjusted for all effect modifiers. However, by having IPD we are doing as well as we can. The inconsistency checks and assessment of heterogeneity are ways to check this. We elaborated on the text of the “Limitations” section, both on the limitations themselves, as well as our plans to mitigate them: “The different evidence synthesis methods make weaker or stronger assumptions, but these come down to there being no differences in effect modifiers between the included studies that have not been adjusted for. The more flexible models using IPD are more able to adjust for effect modifiers more effectively, and so make less strong assumptions. The IPDNMA method allows us to assess the main assumptions and also gives flexibility in the extent that the assumptions are applied (40).”

**OTHER CHANGES MADE TO THE MANUSCRIPT**

Apart from addressing the reviewers’ comments, we have also added some refinements we made to the analysis plan while the manuscript was under review, and we have also improved the writing to improve the overall clarity of the manuscript. More specifically, we are now using a different method for data reduction, i.e. choosing which background variables to test, we have specified in which cases pseudo-IPD will be used, and we now have emphasised our plan to conduct component network meta-analysis. We have also changed the title from “Individual *Patient* Data Network Meta-analysis of psychosocial interventions for survivors of intimate partner violence: Study protocol” to “Individual *Participant* Data Network Meta-analysis of psychosocial interventions for survivors of intimate partner violence: Study protocol”.

The reference list has changed slightly, we have added two references (NICE Guidelines Support Group, 2021 and Chaimani et al, 2013) to explain our choice of method for synthesising different measures of the same outcome and for the assessment of publication bias. The numbering of the references has also changed, given the revisions in the text.

Thank you once again for the consideration.

Kind regards,

Christina Palantza

Karen Morgan

Nicky Welton

Hannah Micklitz

Lasse Sander

Gene Feder
