# Supplementary material for: Individual participant data network meta-analysis of psychosocial interventions for survivors of intimate partner violence: Study protocol
Source: PLoS One. 2025 Mar 18;20(3):e0306669. doi: 10.1371/journal.pone.0306669 (PMC11957554; doi:10.1371/journal.pone.0306669)
Supplement: Attachment — Submitted filename: Response to reviewers Feb25.docx [file pone.0306669.s003.docx]

Dear Dr Leight and dear reviewers,

Thank you very much for thoroughly reviewing the updated version of our manuscript and for picking up on details to ensure that the manuscript is flawless. Below is a point by point reply to all the comments:

Reviewer 1

*“The authors have focused on effectiveness rather than efficacy. I just wanted to confirm/clarify this choice, given that efficacy is usually reported by studies under idealised, research study conditions and (given the inclusion criteria) I would have thought that they will identify evidence like this rather than real-world effectiveness data.”*

We thank the reviewer for the opportunity to better justify our choice to use the term effectiveness instead of efficacy. Based on the criteria described by [Gartlehner et al in 2006](https://www.ncbi.nlm.nih.gov/books/NBK44028/), we are confident that our study measures the effectiveness rather than the efficacy, because:

a) the population is usually recruited from general healthcare or even community settings
b) the eligibility criteria are typically not very refined
c) the outcome criterion varies a lot across trials as subjective, objective and health outcomes have been measured by many of the trials.
d) The study lengths are typically closer to the length of treatment in a clinical setting, and compliance/adherence to the intervention is an outcome measure in our study
e) Adverse events have been reported by less than half of the trials, so this criterion is unclear
f) Sample sizes also differ greatly, so this criterion is unclear too
g) Many of the studies have followed the intention-to-treat principle

In addition, the eligibility criteria of our study allow the inclusion of any control condition, and most of these control conditions are standard/routine care, also commonly referred to as “treatment-as-usual”.

*“References to the Micklitz review have been moved up in the Introduction but as a result the first reference to that paper sounds like readers are assumed to be familiar with it. The phrasing could be tweaked so that the first time the Micklitz paper is mentioned, it summarises what that paper is and how it relates to this study.”*

We thank the reviewer for pointing this out, we have now rephrased the sentence to make sure the Mickplitz paper is introduced properly:

*“*Micklitz and colleagues in their meta-analysis, which is one of the most recent and comprehensive meta-analyses on IPV and mental health, categorised the tested psychosocial interventions into three main categories: psychological, advocacy-oriented and integrated (a combination of the two) (16).”

*“Although the title has changed to individual participant instead of patient, the term patient has persisted, e.g. line 140 - this should be harmonised across the manuscript.”*

We thank the reviewer for picking up this important detail. We have now replaced the word “patient” with that of “participant”, except for the term “Patient and public involvement”, because this is a standard term.

“*There are a few errors arising from the corrections e.g. line 166 "we will fulfil this aim by to conducting...", line 377 "componentss", "studies with aggregate data studies*"”

We thank the reviewer for spotting these mistakes. We have now corrected them.

*“The tense changes at times between present and future, e.g. Table 1 says both "studies are deemed partially eligible" but also "will not be included".”*

We thank the reviewer for stressing this issue, we have now made sure the tense use is consistent.

“*I believe the term used by GRADE is certainty "of" the evidence rather than "in".”*

We thank the reviewer for picking up another important detail. We have corrected it throughout the manuscript.

*“Line 162-165: this sentence is long and the meaning of the phrase "moderators to that" was not clear to me.”*

We thank the reviewer for raising this point. We have now rephrased this sentence to:

“This study aims to provide more valid and detailed estimates of the comparative clinically relevant benefits and negative effects of each type of psychosocial interventions than the existing pairwise meta-analyses. The IPD of the studies will be obtained to enable this estimation, as well as exploration and adjustment of effect modifiers.”

*“Line 384-388 - this is a long sentence with several clauses that could be broken into two to aid clarity.”*

We thank the reviewer for giving us the opportunity to clarify even further. We have rephrased the sentence to:

“We will also create pseudo-IPD by simulating from a model using the reported baseline summaries and regression coefficients for effect modifiers as described in (47), if available in the publications of studies that do not provide IPD.”

Reviewer 2

“*Thank you for responding to my queries to my overall satisfaction. While it is good to be flexible and improve the study design, any changes once the study has begun/been funded should be listed under ‘changes to protocol’ to be compared with the original, as a key reason for publishing protocols, for audit. The editor may wish to follow this point up. Good luck with the analysis and data collection*.”

We thank the reviewer for reminding us and the journal of this crucial aspect. We now include a section listing these changes and we mention the shift in the data reduction method and the increased focus on the comparison of components:

“Changes to the protocol: Since the awarding of the funding and the beginning of the study, a few changes have been made to the protocol. The data reduction method has changed from a lasso method to assessment of collinearity, and more emphasis has been placed on the comparison of intervention components, as it appears to be important for the overall objective of disentangling effectiveness.”

We are confident that these revisions render the protocol ready for publication.

Kind regards,

Christina Palantza
